# Supplementary material for: Mass-sensitive particle tracking to elucidate the membrane-associated MinDE reaction cycle
Source: Nat Methods. 2021 Oct 4;18(10):1239–46. doi: 10.1038/s41592-021-01260-x (PMC8490154; doi:10.1038/s41592-021-01260-x)
Supplement: Supplementary file 1 — Supplementary Figs. 1–20, Discussion, Tables 1–7 and video captions. [file 41592_2021_1260_MOESM1_ESM.pdf]

nature portfolio
https://doi.org/10.1038/s41592-021-01260-x

Supplementary information

Mass-sensitive particle tracking to elucidate the membrane-associated MinDE reaction cycle

In the format provided by the authors and unedited

Supplementary Information

Title

Mass-sensitive particle tracking to elucidate the membrane-associated MinDE reaction cycle

Author List

Tamara Heermann[1,#], Frederik Steiert[1,2,#], Beatrice Ramm[1,3], Nikolas Hundt[4,#,*] and Petra Schwille[1,*]

# equal contribution

Affiliations

[1]Department of Cellular and Molecular Biophysics, Max Planck Institute of Biochemistry, Am Klopferspitz 18, 82152 Planegg, Germany

[2]Department of Physics, Technical University Munich, 85748 Garching, Germany.

[3]Department of Physics, Princeton University, 08544 Princeton, NJ, USA

[4]Department of Cellular Physiology, Biomedical Center (BMC), Ludwig-Maximilians-Universität München, Großhaderner Str. 9, 82152 Planegg, Germany

* corresponding authors:

Prof. Petra Schwille
ORCID: 0000-0002-6106-4847
schwille@biochem.mpg.de
Phone: +49 89 8578-2900
Fax: +49 89 8578-2903

Dr. Nikolas Hundt
ORCID: 0000-0001-8217-671X
nikolas.hundt@med.uni-muenchen.de
Phone: +49 89 2180 71563
Fax: +49 89 2180 71732

Supplementary Figures

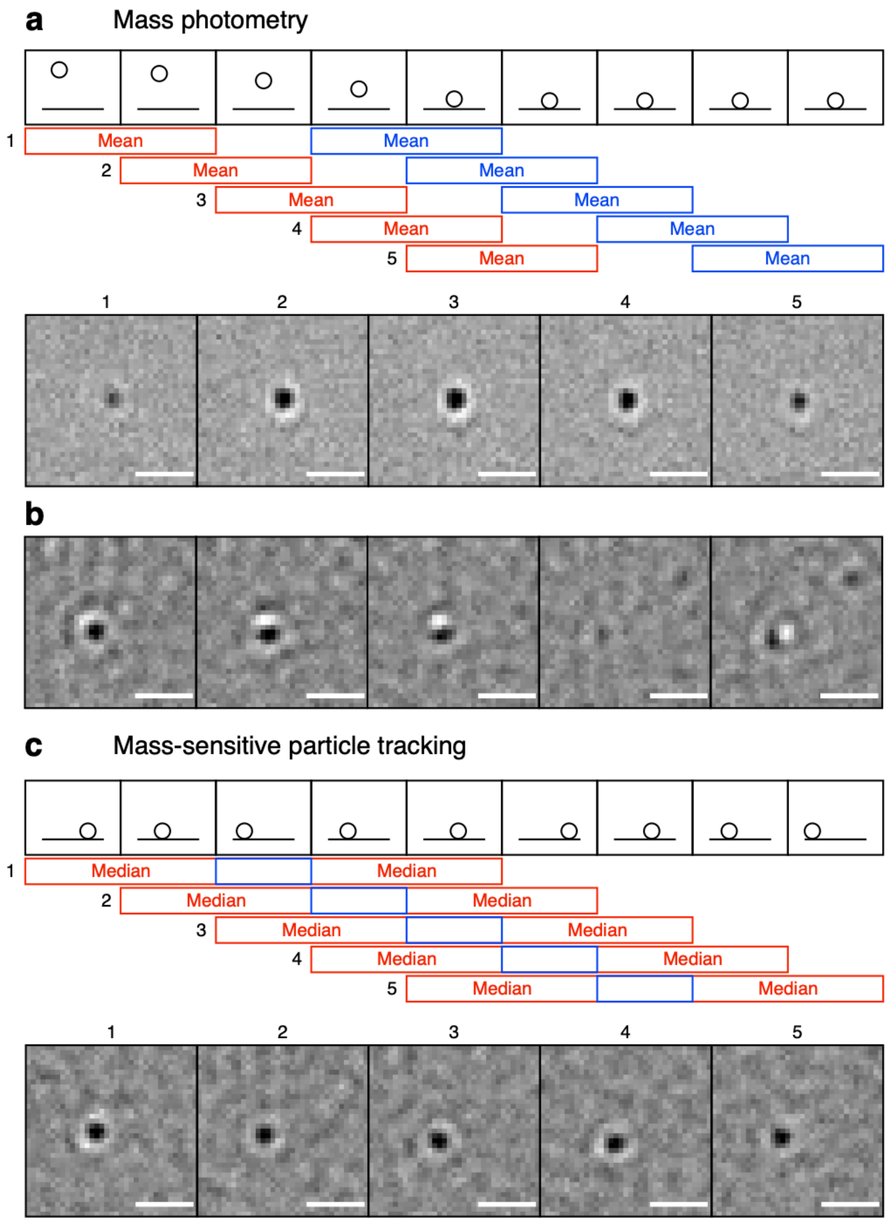

Supplementary Figure 1 - Illustration and comparison of image processing procedures. (a) In mass photometry (MP), landing molecules are visualized through gradually fading their PSF in and out of the video. Top: Schematic representation of a frame sequence of a landing particle. Middle: Mean of pixel intensities of blue frames is divided by the mean pixel intensities of red frames. Bottom: Representative ratiometric frames obtained for a landing event of β-amylase on glass applying this image-processing approach. **(b)** Representative ratiometric frames obtained for a biotin-aldolase-streptavidin complex diffusing on a biotinylated supported lipid bilayer using the processing scheme in (a). Note the distorted and occasionally disappearing PSF. **(c)** For mass-sensitive particle tracking (MSPT), mobile features are visualized by removing background that contains only static features. Top: Schematic representation of a frame sequence of a randomly moving particle on a surface. Middle: Pixel intensities of the blue central frame are divided by the median of pixel intensities of surrounding red frames. Bottom: Representative ratiometric frames obtained for the same biotin-aldolase-streptavidin complex as in (b) using the image-processing approach in (c). Scale bars: 1 µm.

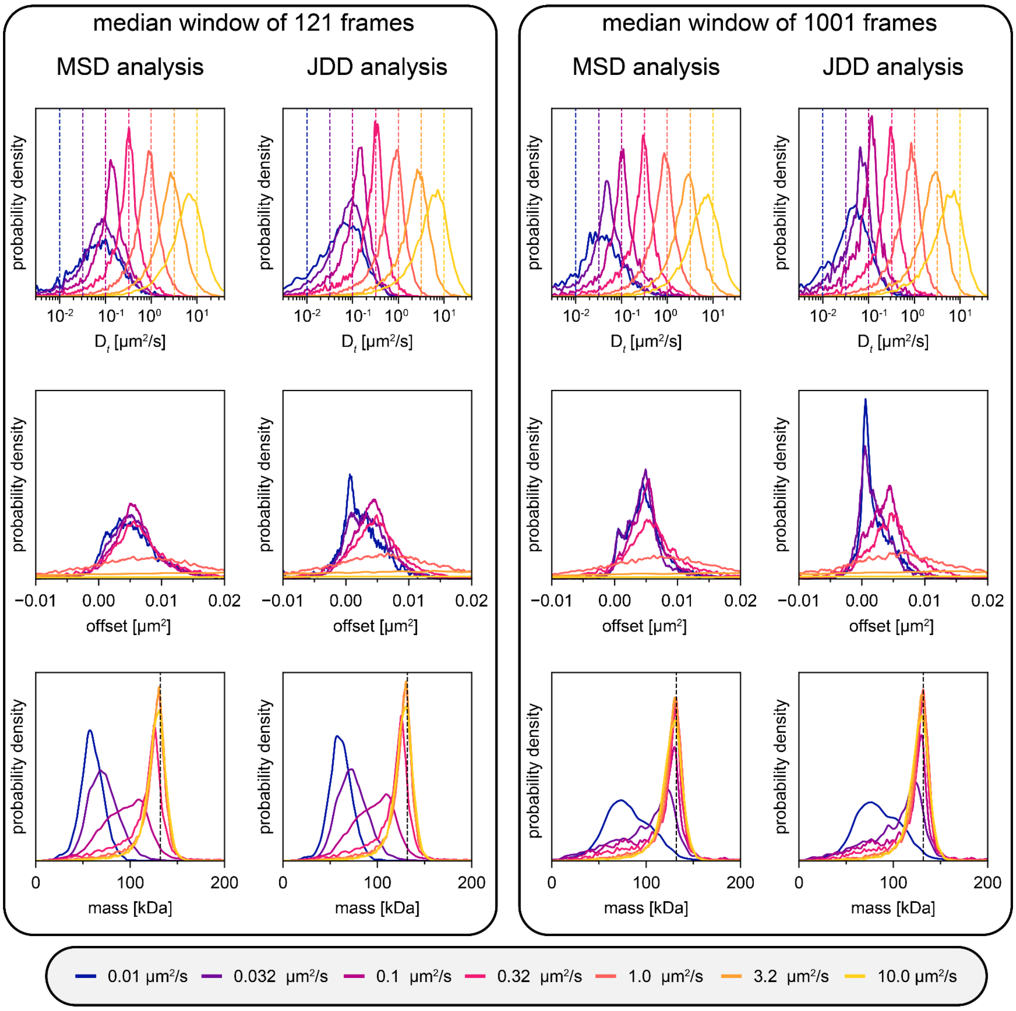

Supplementary Figure 2 – Comparison of mean squared displacement (MSD) and jump-distance distribution (JDD) analysis performance using simulated MSPT videos with varying diffusion coefficients. Both the left and right panel each display the distribution of determined diffusion coefficients (top row), the fitted offset corresponding to the particle localization uncertainty (middle row) and the extracted mass distribution (bottom row) for MSD and JDD analysis. Simulated videos used for particle tracking were processed either with a median window of 121 frames (left panel) or with a median window of 1001 frames (right panel). In the top row, dashed lines indicate the input diffusion coefficient and the distributions in the corresponding color show the diffusion coefficients extracted from the respective simulated videos. In the bottom row, the dashed line indicates the input mass (i.e. input particle contrast). The average particle density in the videos varied between 0.06 and 0.16 µm^{-2}. For the number of analyzed trajectories, please refer to Supplementary Table 5.

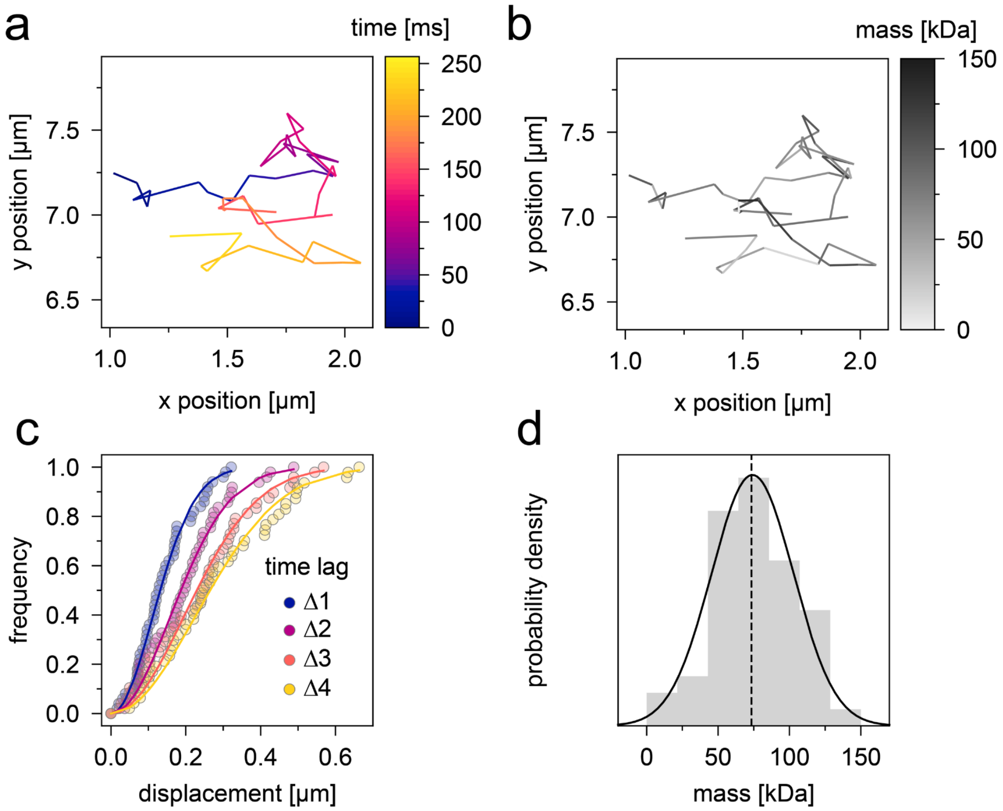

Supplementary Figure 3 - Single-particle characterization of the diffusion of membrane-attached streptavidin by MSPT. (a, b) Representation of a reconstructed particle trajectory color-coded by time (**a**) or mass (**b**). **(c)** Cumulative distribution of displacements for time lags from Δ1-4 frames globally fitted with an equation describing two-dimensional Brownian motion (see methods). **(d)** Histogram of the masses detected in each frame along the trajectory (grey) and its approximation with a normal distribution (black line). The dashed line represents the median.

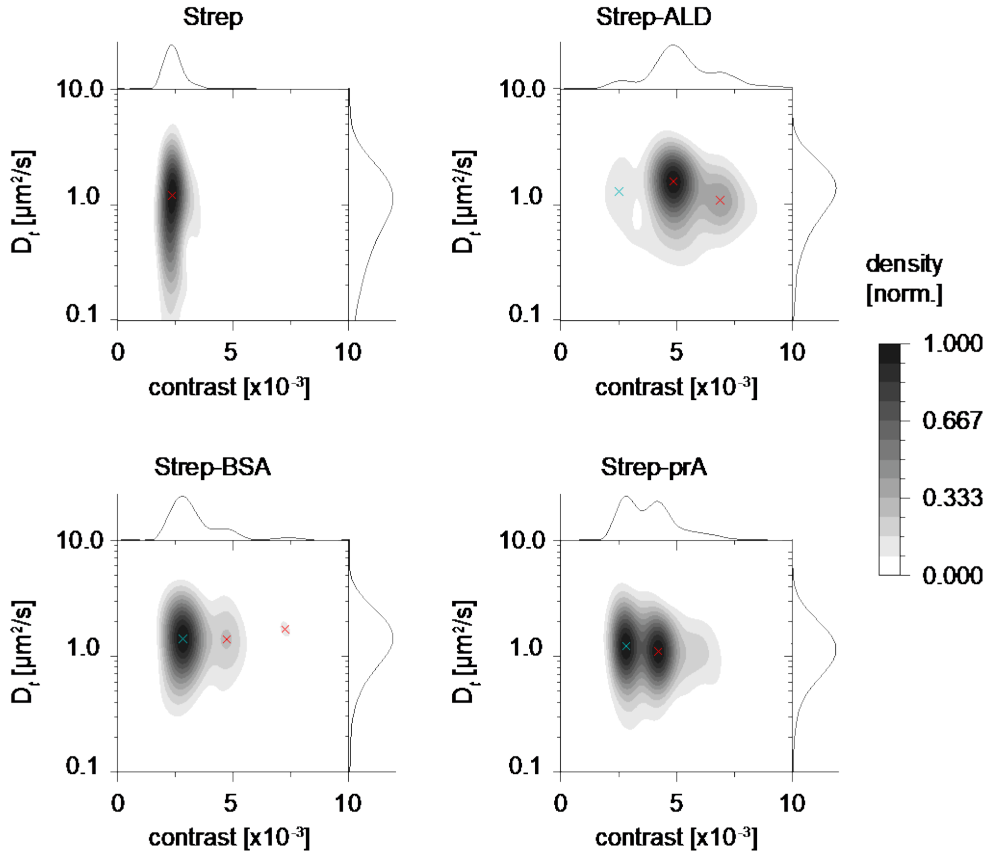

Supplementary Figure 4 - 2D maps of diffusion coefficient and mass for biotinylated MSPT standard proteins attached via divalent streptavidin to biotinylated lipids on a supported lipid bilayer. Only particles with a track length of at least 10 frames were included. Oligomer states included in the MSPT mass calibration are highlighted with a red x. A turquoise x marks peaks that were not considered for mass calibration because they represent unbound divalent streptavidin. Trajectory numbers: divalent streptavidin, Strep, n = 16,699; divalent streptavidin with biotinylated aldolase, Strep-ALD, n = 16,727; divalent streptavidin with biotinylated bovine serum albumin, Strep-BSA, n = 8,842 and divalent streptavidin with biotinylated protein A, Strep-prA, n = 22,424. Marginal probability distributions of both molecular mass (top) and diffusion coefficient (right) are presented.

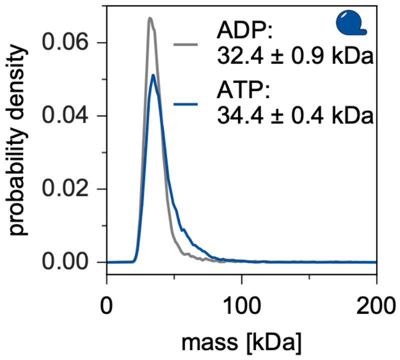

Supplementary Figure 5 - The MinD monomer state is preserved in solution. Mass distributions of 175 nM MinD in the presence of 0.5 mM ATP (blue line; n = 16,013 particles) or 0.5 mM ADP (grey line; n = 7,001 particles) determined by mass photometry.

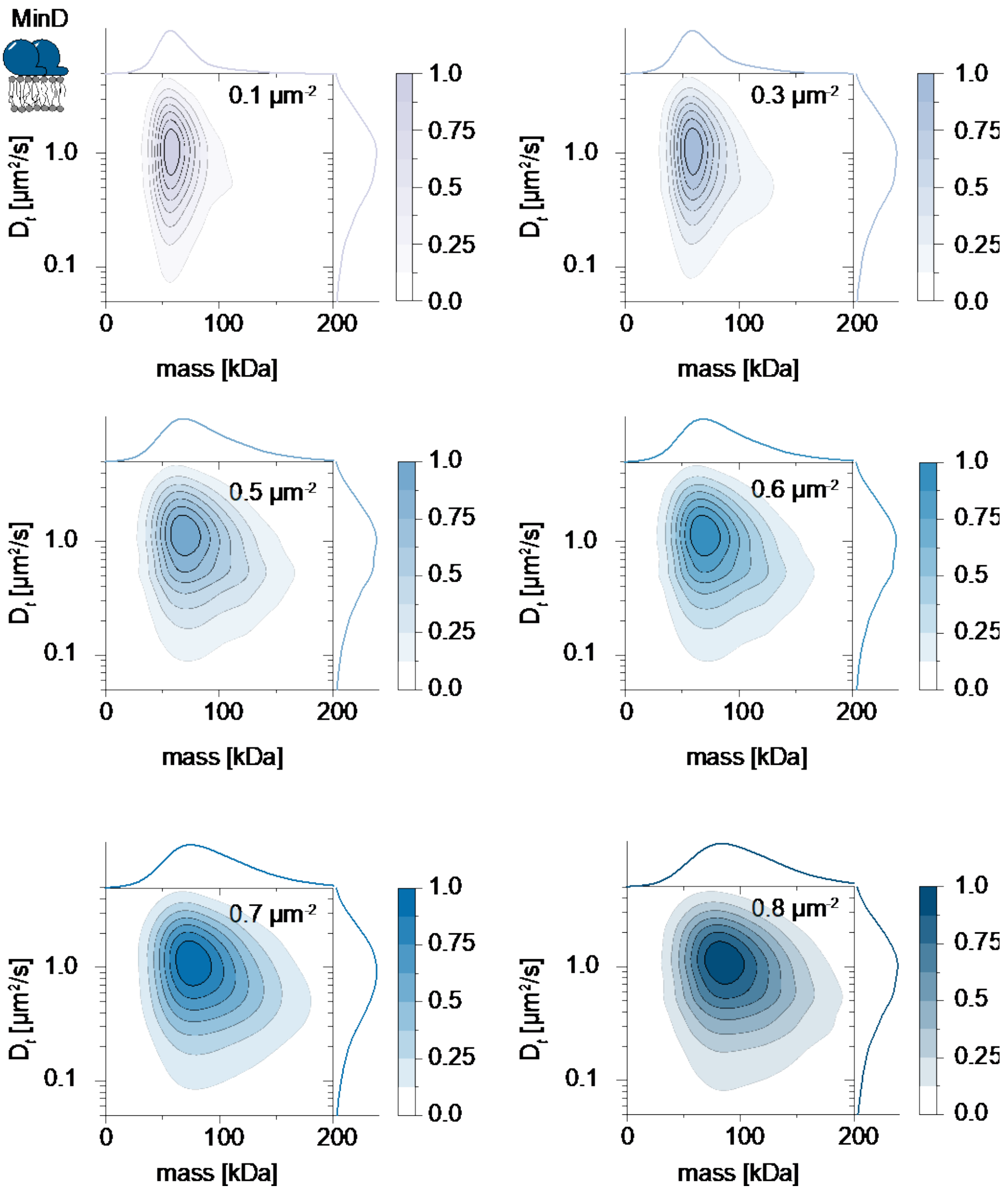

Supplementary Figure 6 - 2D maps of diffusion coefficient and mass for membrane-bound MinD particles. 2D kernel density estimation of diffusion coefficient versus mass for membrane-attached MinD at particle densities of 0.1 µm^{-2} (light mauve, n = 117,086 trajectories), 0.3 µm^{-2} (mauve, n = 169,957 trajectories), 0.5 µm^{-2} (light blue, n = 256,404 trajectories), 0.6 µm^{-2} (blue, n = 256,404 trajectories), 0.7 µm^{-2} (dark blue, n = 282,414 trajectories) and 0.8 µm^{-2} (midnight blue, n = 152,685 trajectories). Marginal probability distributions of both molecular mass (top) and diffusion coefficient (right) are presented.

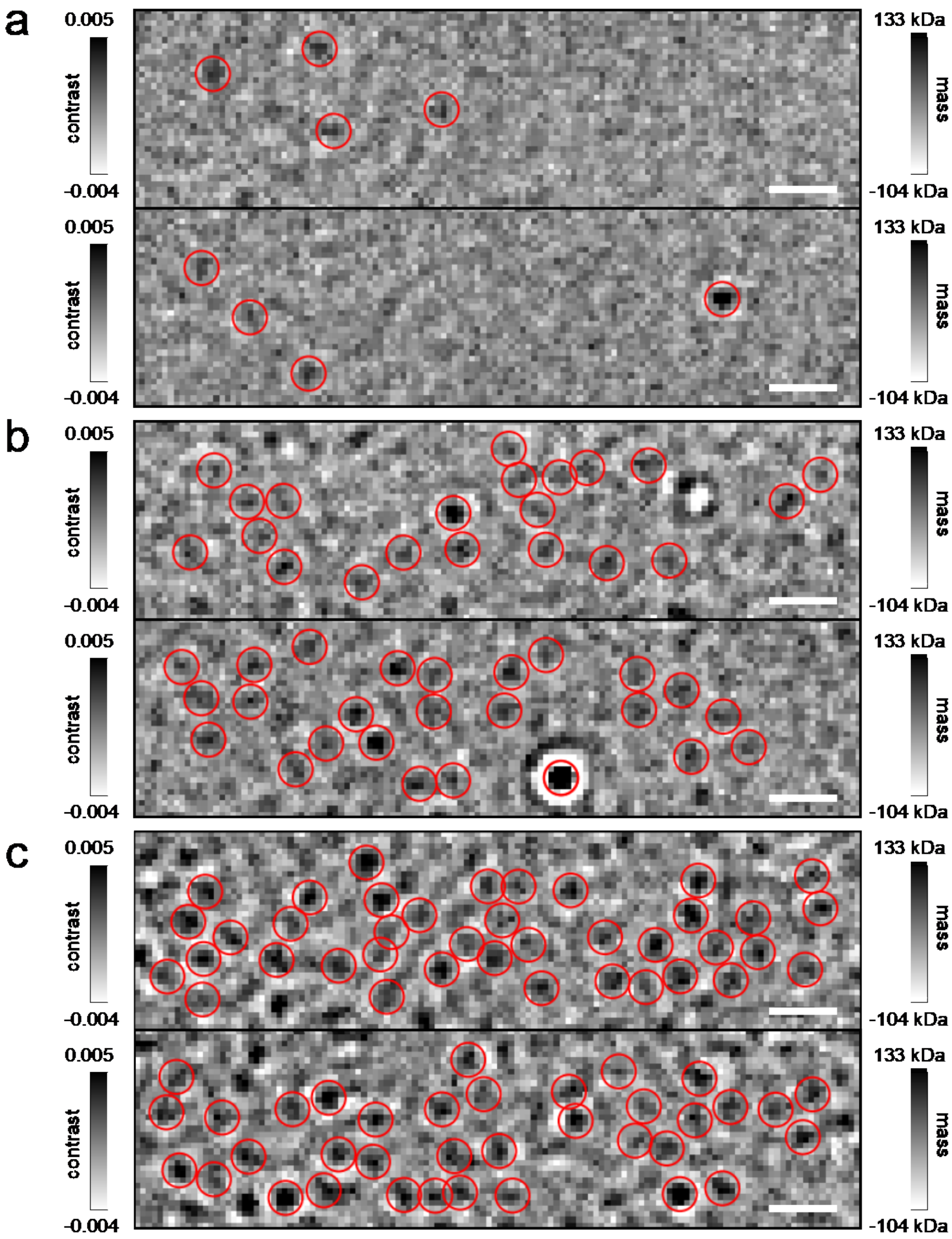

Supplementary Figure 7 - Representative iSCAT images of diffusing MinD particles on a supported lipid bilayer at three different particle densities. Detected and fitted MinD particles (i.e. localized particles) are highlighted through red circles at three different particle densities, 0.1 μm^{-2} **(a)**, 0.5 μm^{-2} **(b)** and 0.8 μm^{-2} **(c)**. Note that some candidates will be lost in the process of trajectory linking. Contrast range: black, 0.005 ≙ 133 kDa; white, -0.004 ≙ -104 kDa. Scale bars: 1 μm.

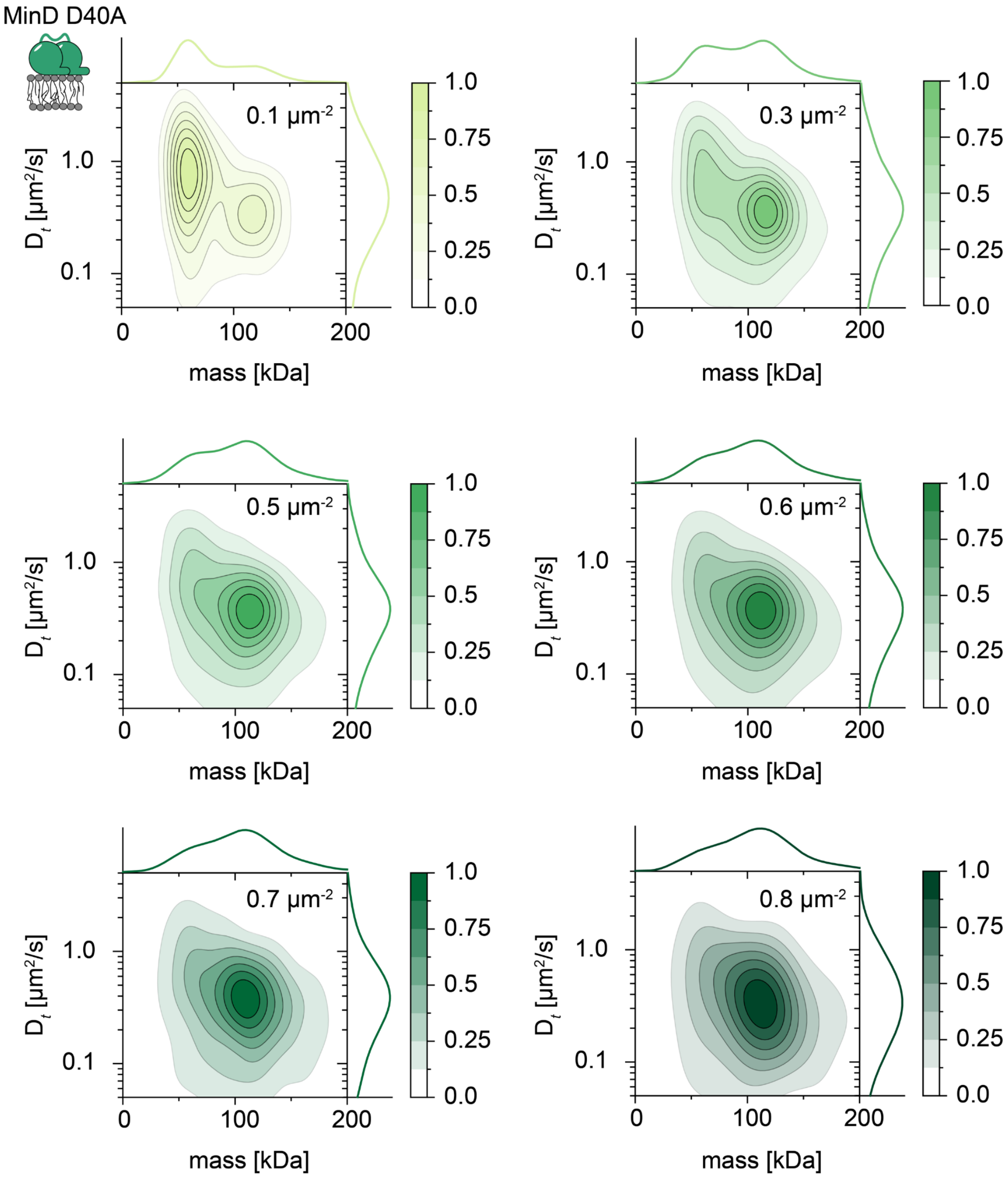

Supplementary Figure 8 - 2D maps of diffusion coefficient and mass for membrane-bound MinD D40A particles. 2D kernel density estimation of diffusion coefficient versus mass for membrane-attached MinD D40A at particle densities of 0.1 µm^{-2} (lime, n = 7,831 trajectories), 0.3 µm^{-2} (fern, n = 72,872 trajectories), 0.5 µm^{-2} (emerald, n = 72,529 trajectories), 0.6 µm^{-2} (sea green, n = 42,280 trajectories), 0.7 µm^{-2} (seaweed, n = 12,172 trajectories) and 0.8 µm^{-2} (viridian, n = 3,150 trajectories). Marginal probability distributions of both molecular mass (top) and diffusion coefficient (right) are presented.

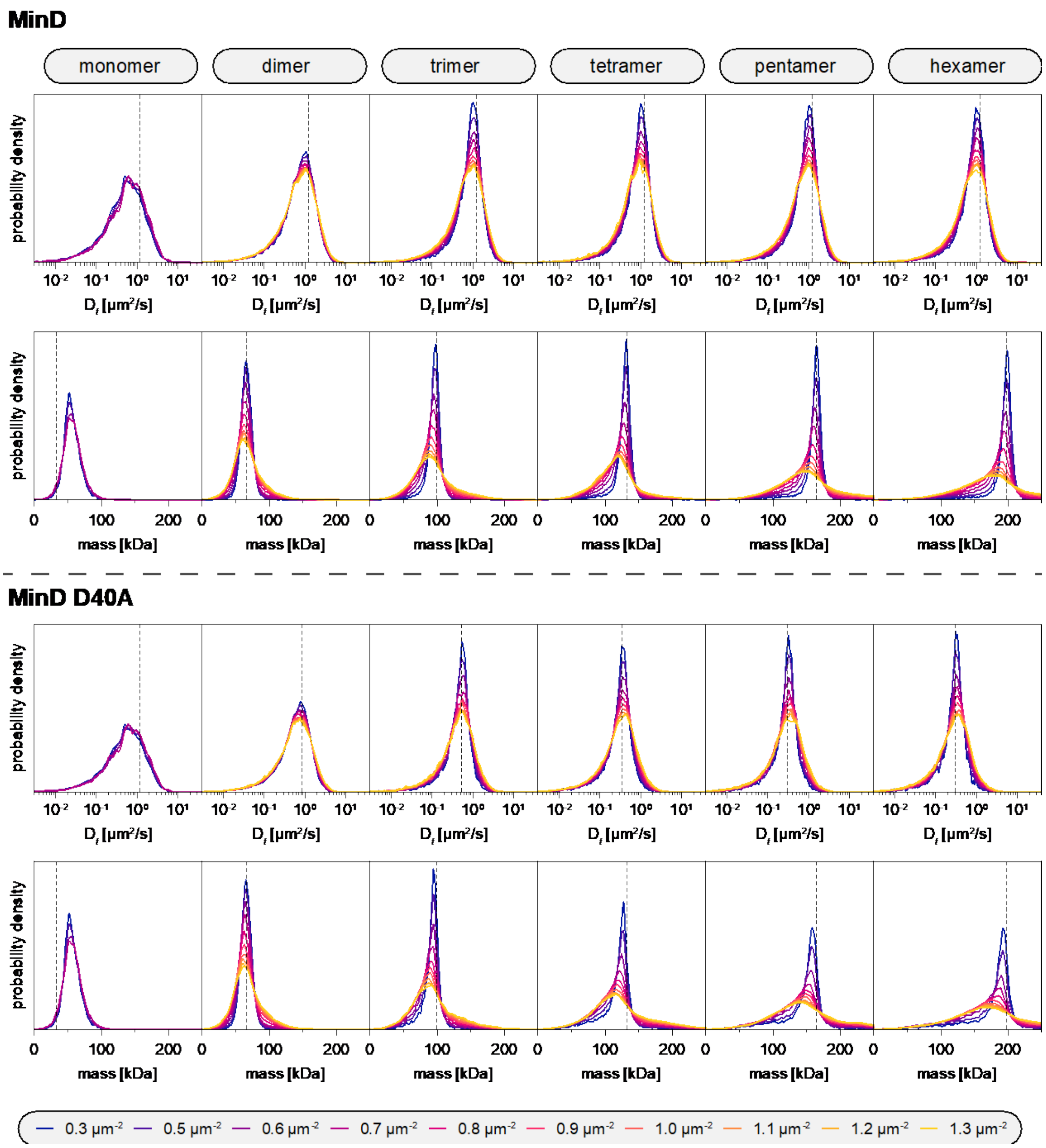

Supplementary Figure 9 – Effect of high particle densities on extracted diffusion coefficient and particle mass in simulated MSPT videos. Distributions of determined diffusion coefficients (top row) and extracted masses (bottom row) for videos with simulated particles corresponding to MinD and MinD D40A oligomers. The simulation was set up with particles that had a constant diffusion coefficient of 1.2 µm^2/s and iSCAT contrasts corresponding to a MinD monomer (33 kDa), a dimer (66 kDa), a trimer (99 kDa), a tetramer (132 kDa), a pentamer (165 kDa) and a hexamer (198 kDa). Simulations of MinD D40A particles were set up with identical masses as for MinD but with a varying diffusion coefficient for monomers (1.2 µm^2/s), dimers (0.85 µm^2/s), trimers (0.54 µm^2/s), tetramers (0.34 µm^2/s) and 0.3 µm^2/s for both penta- and hexamers. Distributions are grouped by localized particle density as a better estimate for the membrane crowdedness in a corresponding experimental video section. We used these oligomer-specific mass distribution shapes for the multicomponent fits in Fig. 2e-h and Supplementary Fig. 11. MinD and MinD D40A monomer detections are not possible at localized particle densities beyond 0.8 µm^{-2}. For the number of analyzed trajectories, please refer to Supplementary Table 6.

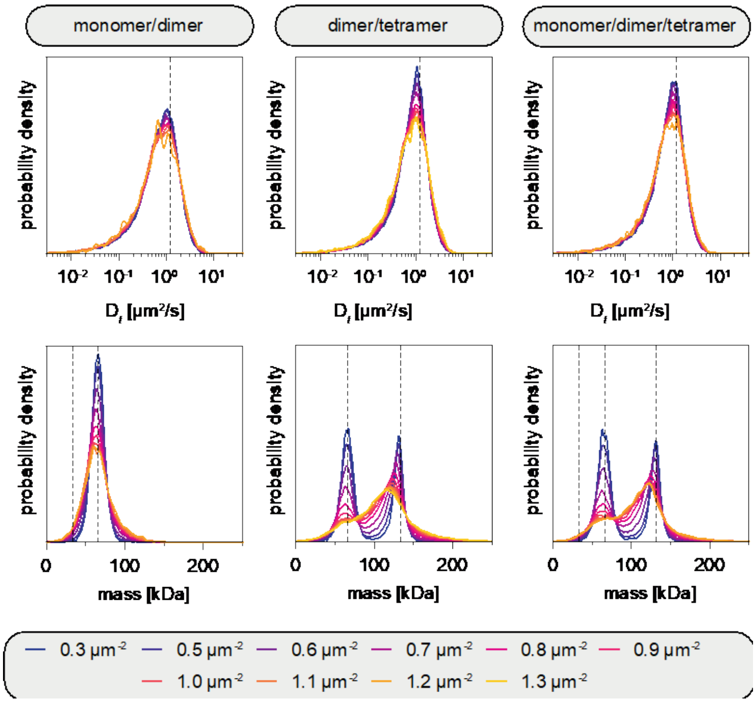

Supplementary Figure 10 – Effect of mixtures of MinD oligomers as observed with simulated MSPT videos. Distributions of determined diffusion coefficients (top row) and the extracted mass (bottom row) for videos containing simulated particles corresponding to mixtures of MinD oligomers. The simulation was set up with particles that had contrasts corresponding to MinD monomers and dimers (33 kDa – monomer, 66 kDa – dimer; left column) in a 1:1 mixture, a 1:1 mixture of MinD dimers and tetramers (132 kDa – tetramer, middle column), and a 1:1:1 composition of particles with the contrasts of a MinD monomer, dimer and tetramer (right column). Distributions are grouped by localized particle density. For the number of analyzed trajectories, please refer to Supplementary Table 7.

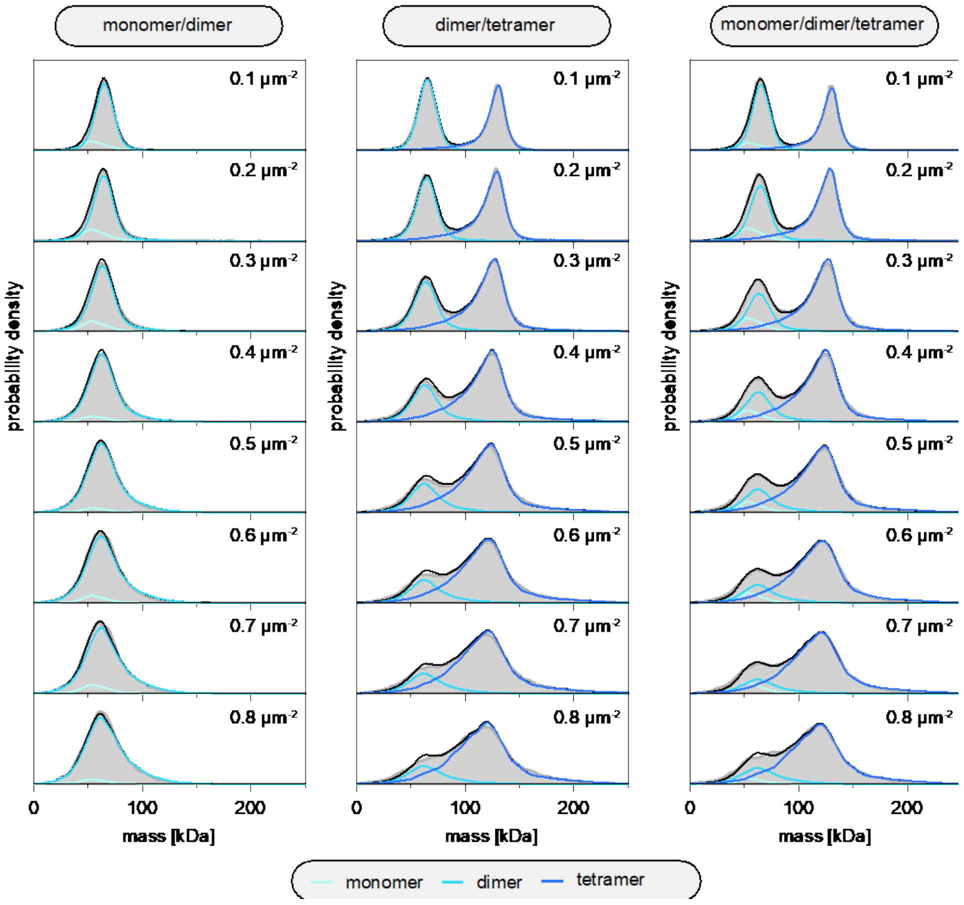

Supplementary Figure 11 – Mass distributions of simulated particle mixtures fitted using separate oligomer components in Supplementary Fig. 9. For each of the three simulated particle mixtures - monomer/dimer, dimer/tetramer, monomer/dimer/tetramer – the representative mass distributions (grey) and corresponding fit (black line, colored lines highlight underlying components) is shown using a linear combination of the underlying separate components for eight different particle densities. For the number of analyzed trajectories, please refer to Supplementary Table 7.

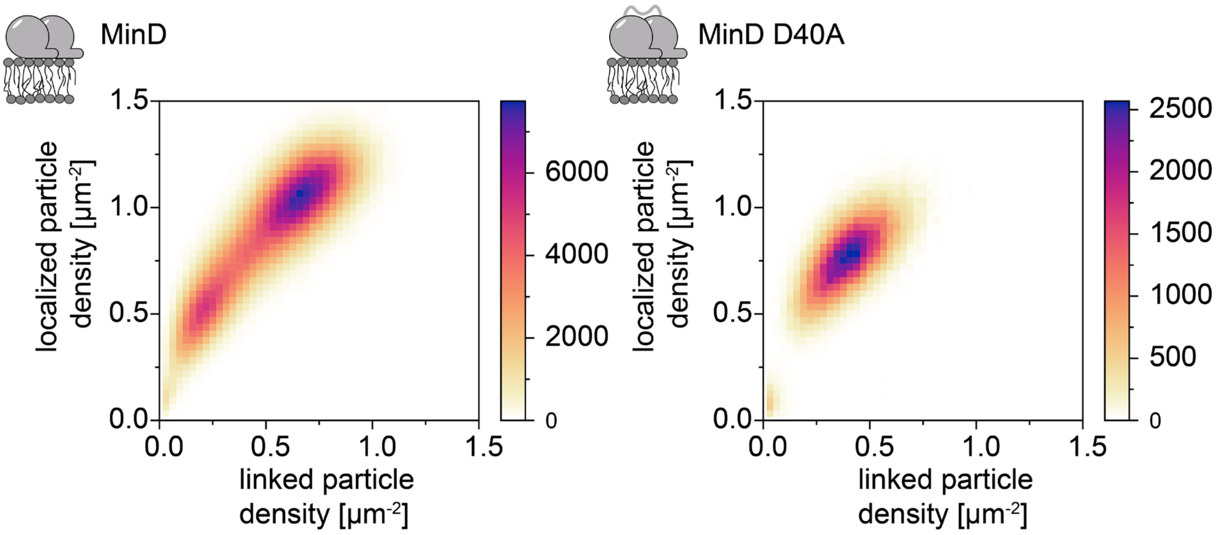

Supplementary Figure 12 – Correlation of localized and linked particle densities for MinD and MinD D40A. Comparison of frame-wise particle localizations per FOV to the linked particle density returned by the MSPT analysis. Note that linked particle densities are reduced by about 40% due to the custom-set trajectory length threshold of five frames and imperfect linking. Linked particle numbers: MinD n = 1,102,940 and MinD D40A n = 194,545.

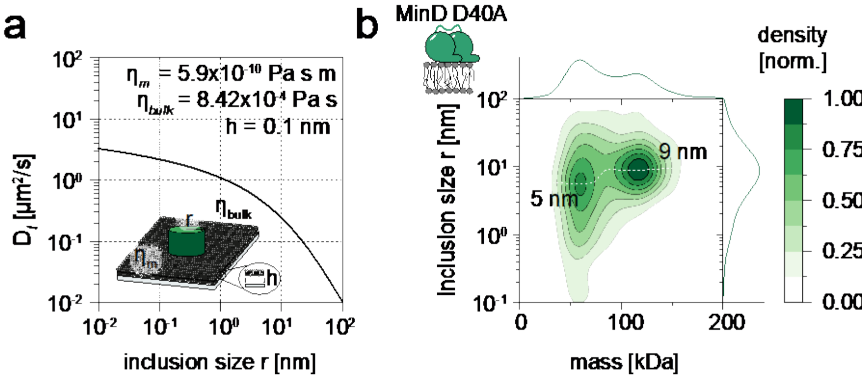

Supplementary Figure 13 - MinD D40A tetramers insert all their subunit MTSs into the bilayer. (a) Theoretical relationship between diffusion coefficient and inclusion size radius of an object in a supported lipid bilayer according to the theory of Evans-Sackmann[1], assuming a similar membrane viscosity (η_m) as for a pure DOPC membrane[2]. We have assumed 8.42×10^{-4} Pa s [2] for the bulk viscosity η_{bulk}, and a height of 0.1 nm for the lubricating layer parameter h according to[1]. **(b)** Analysis of the inclusion size radius as a function of MinD D40A particle mass at a particle density of 0.1 μm^{-2} (n = 12,063 trajectories). The dimer population had an estimated inclusion size radius of 5 nm, whereas the radius of the tetramer was 9 nm, indicating that the tetramer inserts additional MTSs into the bilayer. The white dashed line highlights the shift in inclusion size for increasing particle mass. Marginal probability distributions of both molecular mass (top) and inclusion size radius (right) are presented.

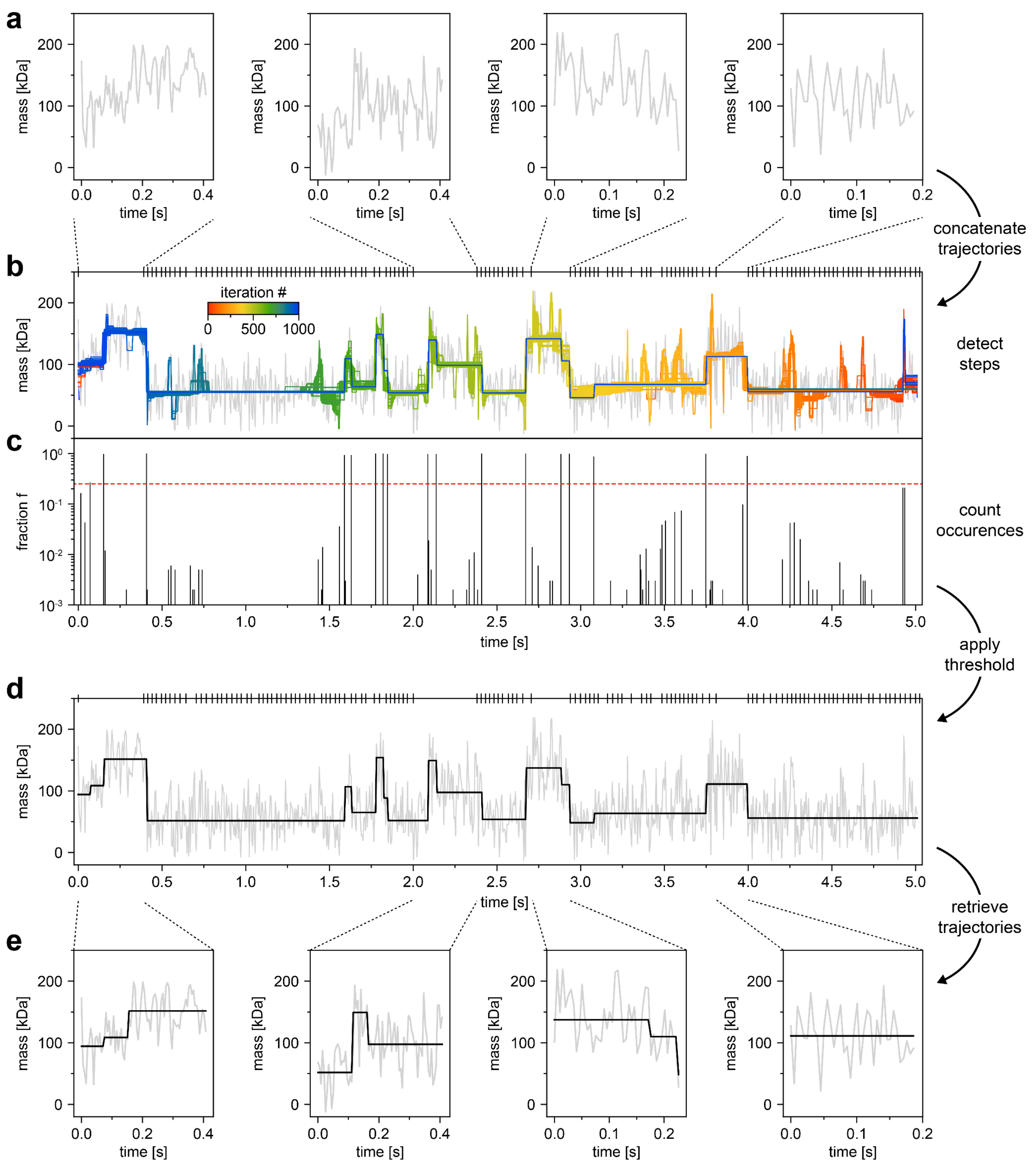

Supplementary Figure 14 – Step detection workflow. (a) Trajectories were concatenated in random order **(b),** beginning and end of individual trajectories is indicated by vertical lines at the top. Steps in subsections with a length of 1000 frames were identified using the Kalafut-Visscher algorithm[3], shifting the start point by one increment in each iteration. The significance of a step is resembled by its frequency of detection **(c)**, displayed as fraction *f* (frequency/1000) in logarithmic scale. Only steps that were detected more frequently than a threshold fraction (0.25) are retained **(d)**. The times series and its resulting step fit was then separated back into the original trajectories **(e)**.

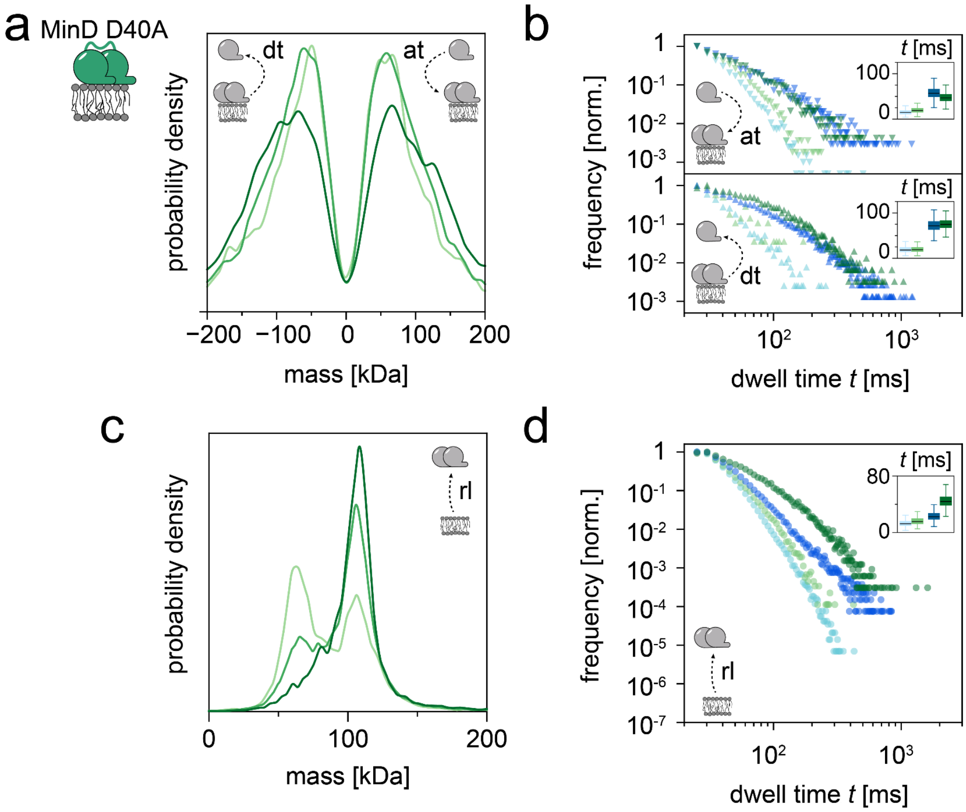

Supplementary Figure 15 – Subunit (dis-)assembly of MinD D40A particles diffusing on a membrane. (a) Mass step size distribution revealing MinD D40A subunit turnover at particle densities of 0.1 μm^{-2} – pale green (n = 4,373 plateaus), 0.3 μm^{-2} – light green (n = 17,443 plateaus), 0.5 μm^{-2} – green (n = 4,746 plateaus). **(b)** Dwell time plots for MinD D40A and MinD subunit attachments (at, top plot) and detachments (dt, bottom plot). Dwell times are shown for the MinD dimer (light blue line) and MinD tetramer (dark blue line) as well as for their respective MinD D40A versions (dimer state – light green, tetramer state – green). Plateau numbers for (at): MinD dimer – n = 23,782, MinD D40A dimer – n = 6,862, MinD tetramer – n = 5,088, MinD D40A tetramer – n = 2,954; (dt): MinD dimer – n = 3,143, MinD D40A dimer – n = 295, MinD tetramer – n = 10,406, MinD D40A tetramer – n = 5,162. Inset: Box plots indicate second and third quantile (box), median (horizontal line), and 1.5-times the interquartile range (whiskers) of bootstrapped mean dwell times (n = 10,000). **(c)** MinD D40A mass distribution for membrane release (rl) events at 0.1 μm^{-2} – pale green (n = 12,063 plateaus), 0.3 μm^{-2} – light green (n = 72,872 plateaus), 0.5 μm^{-2} – green (n = 24,425 plateaus). **(d)** Plot of the dwell times before membrane release for the MinD D40A dimer and tetramer state and the respective MinD versions for comparison. Plateau numbers: MinD dimer – n = 562,011, MinD D40A dimer – n = 42,119, MinD tetramer – n = 73,037, MinD D40A tetramer – n = 28,467. Inset: Box plot details as described in (b).

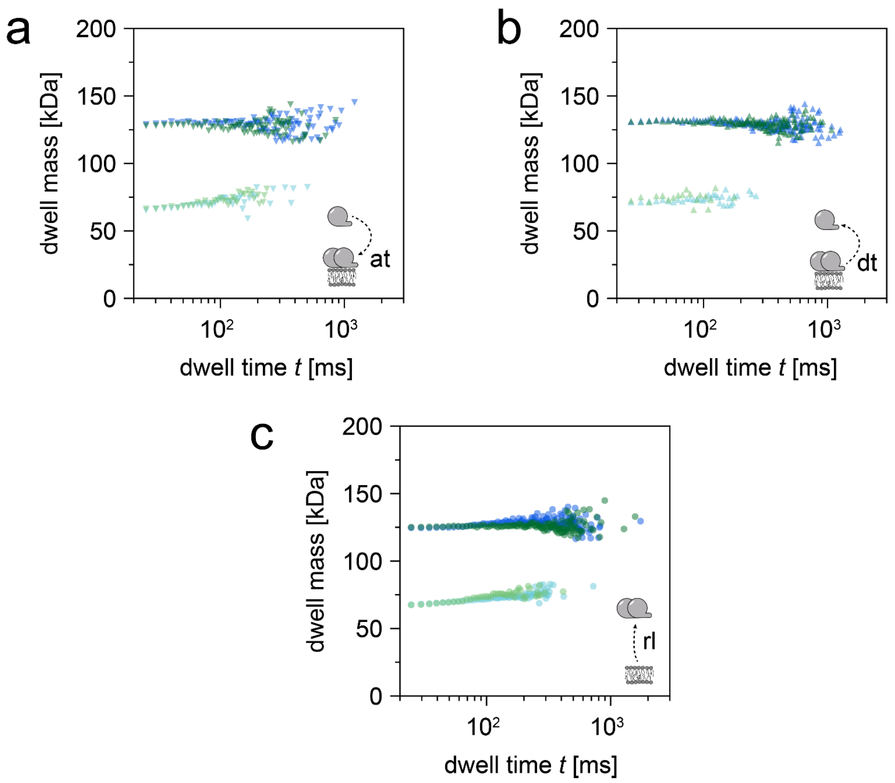

Supplementary Figure 16 - Plots of dwell mass in relation to the dwell time for attachment events (a), detachment events (b) and membrane release (c), to illustrate mass-based categorization of MinD and MinD D40A particles into dimer and tetramer states. Dwell masses are shown for the MinD dimer (light blue) and MinD tetramer (dark blue) as well as for MinD D40A dimer (light green) and MinD D40A tetramer (dark green). **(a)** Plateau numbers for attachment events (at): MinD dimer – n = 23,782, MinD D40A dimer – n = 6,862, MinD tetramer – n = 5,088, MinD D40A tetramer – n = 2,954 **(b)** Plateau numbers for detachment events (dt): MinD dimer – n = 3,143, MinD D40A dimer – n = 295, MinD tetramer – n = 10,406, MinD D40A tetramer – n = 5,162. **(c)** Plateau numbers for release events (rl): MinD dimer – n = 562,011, MinD D40A dimer – n = 42,119, MinD tetramer – n = 73,037, MinD D49A tetramer – n = 28,467.

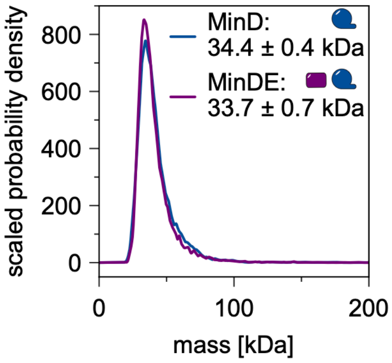

Supplementary Figure 17 - In the absence of the catalytic membrane interface, MinD and MinE do not interact. Probability density scaled by the number of detected molecules in videos of the same length for the mass distribution of 175 nM MinD (blue line; n = 16,013 particles), 175 nM MinDE (magenta line; n = 15,399) in the presence of 0.5 mM ATP.

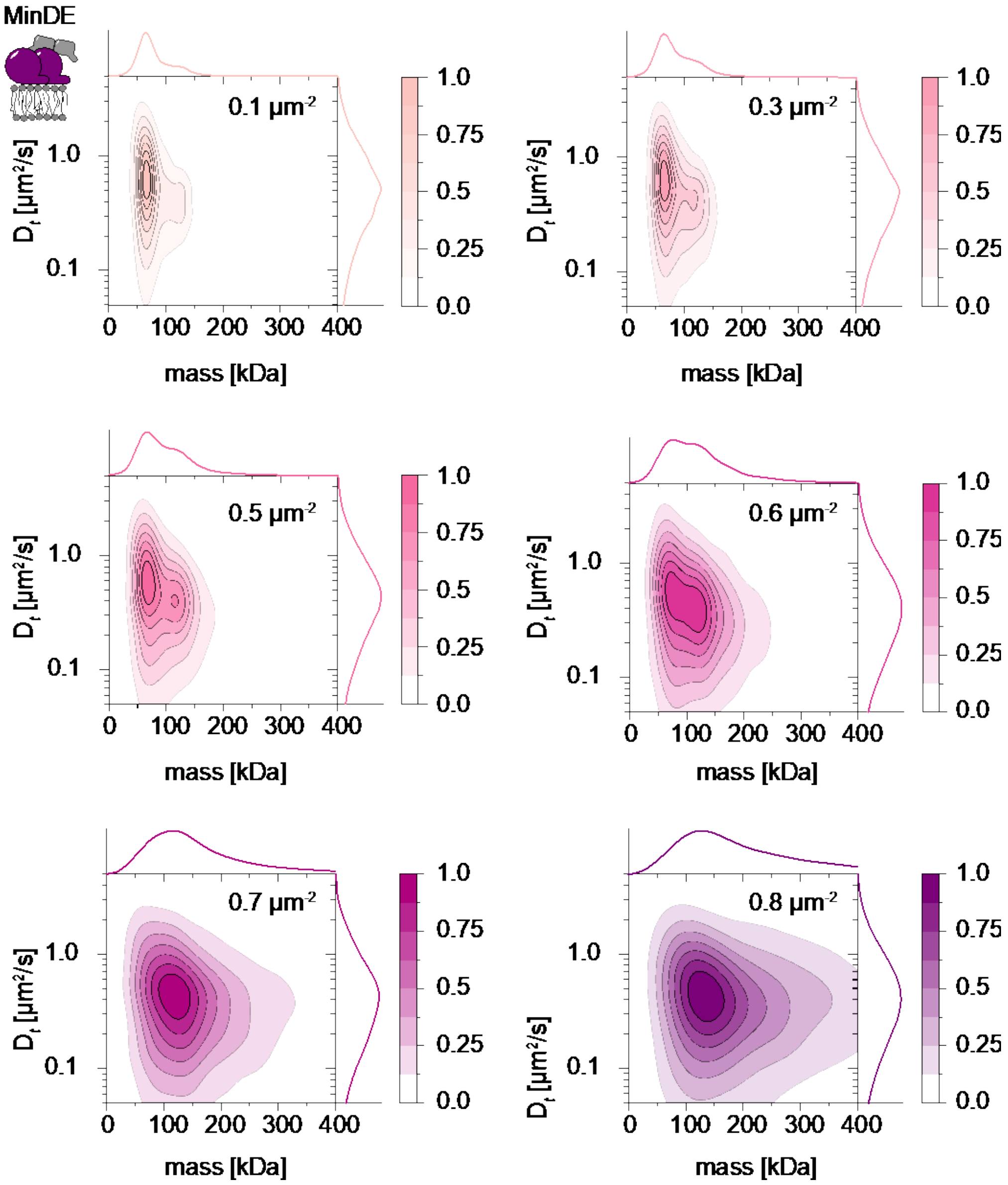

Supplementary Figure 18 - 2D maps of diffusion coefficient and mass for membrane-bound MinDE particles. 2D kernel density estimation of diffusion coefficient versus mass for membrane-attached MinDE at particle densities of 0.1 µm^{-2} (light mauve, n = 200,436 trajectories), 0.3 µm^{-2} (mauve, n = 158,660 trajectories), 0.5 µm^{-2} (light pink, n = 35,501 trajectories), 0.6 µm^{-2} (pink, n = 35,885 trajectories), 0.7 µm^{-2} (light purple, n = 36,293 trajectories) and 0.8 µm^{-2} (purple, n = 30,083 trajectories). Marginal probability distributions of both molecular mass (top) and diffusion coefficient (right) are presented.

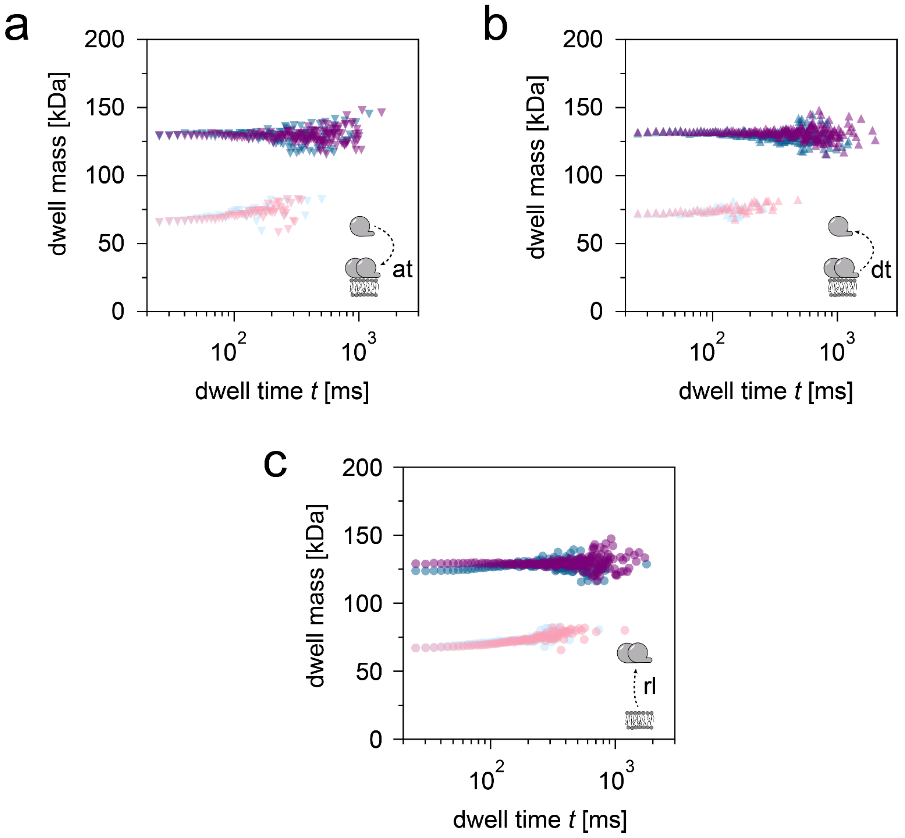

Supplementary Figure 19 - Plots of dwell mass in relation to the dwell time for attachment events (a), detachment events (b) and membrane release (c), to illustrate mass-based categorization of MinDE (pink shades) and MinD (blue shades) particles into dimer and tetramer states. Dwell masses are shown for the MinD dimer (light blue) and MinD tetramer (dark blue) as well as for their respective MinDE complex versions (dimer state – light pink, tetramer state – pink). **(a)** Plateau numbers for attachment events (at): MinD dimer – n = 23,782, MinDE dimer – n = 37,278 and MinD tetramer – n = 5,088, MinDE tetramer – n = 11,698. **(b)** Plateau numbers for detachment events (dt): MinD dimer – n = 3,143, MinDE dimer – n = 3,974 and MinD tetramer – n = 10,406, MinDE tetramer – n = 22,501. **(c)** Plateau numbers for release events (rl): MinD dimer – n = 562,011, MinDE dimer – n = 277,782 and MinD tetramer – n = 73,037, MinDE tetramer – n = 60,114.

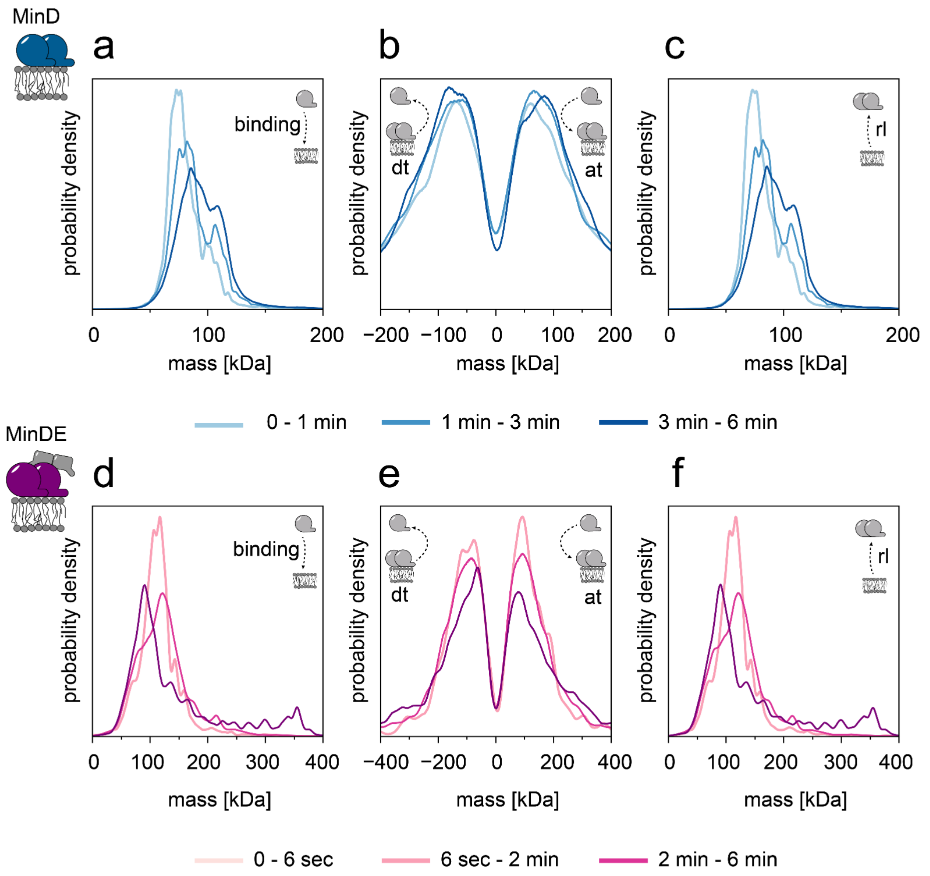

Supplementary Figure 20 – Time-dependence of the membrane-associated behavior of MinD and MinDE. Plots depict the evolution of MinD (blue shades) and MinDE (purple shades) mass distributions for different time segments of a recorded video at a particle density of 0.5 μm^{-2}. MinD time segments: light blue – 0 to 1 min; blue – 1 to 3 min; dark blue – 3 to 6 min. MinDE time segments: light purple – 0 to 6 sec; purple – 6 sec to 2 min; dark purple – 2 min to 6 min. **(a, d)** MinD and MinDE mass distribution for membrane binding events. Plateau numbers for MinD (from light to dark blue): 53,176; 92,227; 139,510 and for MinDE (from light to dark purple): 5,161; 17,805; 12,535. **(b, e)** Mass step size distribution revealing the time-resolved subunit turnover of MinD and MinDE particles. Number of events for MinD (from light to dark blue): 4,630; 8,324; 12,910 and for MinDE (from light to dark purple): 1,087; 4,926; 3,234. **(c, f)** MinD and MinDE mass distribution for membrane release (rl) events. Number of plateaus for MinD (from light to dark blue): 53,176; 92,227; 139,510 and for MinDE (from light to dark purple): 5,161; 17,805; 12,535.

Supplementary Discussion

For readers interested in applying the MSPT routine to their own iSCAT movies, we recommend to consider the details outlined in this Supplementary Discussion. As described in the methods section "Simulated videos of diffusing particles", we set up a simulation that generates artificial videos of diffusing particles on an experimental SLB background. This simulation enabled us to test the performance of our MSPT analysis routine for different membrane scenarios.

We first assessed how well our analysis pipeline could reproduce the diffusion coefficient of particles with a size corresponding to MinD tetramers undergoing Brownian motion. By visual inspection of the videos, a median window size of 121 frames seemed sufficient to visualize particles diffusing at around 1 $\mu m^2 s^{-1}$, which was on the order of magnitude expected in our experimental videos. As pointed out earlier, the median-based background estimation works only if particles do not dwell for too long in a certain area. Therefore, we expected that the method would fail for lower diffusion coefficients. In fact, MSPT reproduced the input diffusion coefficients well between 0.32 $\mu m^2/s$ and 10 $\mu m^2/s$. However, below 0.32 $\mu m^2/s$, coefficients were systematically overestimated (Supplementary Fig. 2, left box, top row). The reason for this phenomenon becomes apparent when considering the extracted mass distributions (Supplementary Fig. 2, left box, bottom row). Since local background estimates get increasingly biased towards the value of background plus particle, if particles remain locally confined for the majority of the median period, the particles loose contrast at low diffusion coefficients. Consequently, masses were underestimated by the MSPT routine at diffusion coefficients below 0.32 $\mu m^2/s$ and gradually fell below the detection level. Thus, the overestimation of slow diffusion coefficients is probably caused by fast particles with a higher SNR being more likely detected than the comparably slower ones with reduced SNR in the ensemble of simulated particle speeds.

The problem of particle confinement for the background estimation can be partially overcome by expanding the size of the median window. As shown in Supplementary Fig. 2, right box, both diffusion coefficient and particle mass were correctly extracted down to a diffusion speed of 0.1 $\mu m^2/s$, if a median size of 1001 frames was chosen. Therefore, we recommend to carefully check that the median window size is chosen appropriately for the speed range of the imaged particles. Note, however, that large median windows drastically affect the image processing speed of the analysis routine and may become sensitive to sample drift.

We also compared the performance of two of the most common methods to extract diffusion coefficients from single-particle trajectories, namely MSD and JDD analysis (Supplementary Fig. 2). We found that MSD performed slightly better, when extracting diffusion coefficients of slowly diffusing particles. However, for our experimental data with faster particles but many short trajectories we found that JDD produced narrower diffusion coefficient distributions. Therefore, we decided to employ this analysis method throughout the rest of the study.

In another scenario, we investigated the consequences of a crowded bilayer for our MSPT analysis. Similar to the confined particle scenario, we expected that the median-based background estimation would be affected, if many particles on the bilayer increased the probability that a location remains occupied by diffusing particles with diffusion coefficients between 0.3 to 1.2 $\mu m^2/s$. Again, we compared the outcome for the distributions of diffusion coefficient and mass with the simulation inputs (Supplementary Fig. 9 and 10). While the

estimated diffusion coefficients remained correct, we observed an increasing distortion of the mass distributions towards low masses at high particle densities. This effect was most pronounced for particles with high contrast. Dense bilayers probably lead to a similar situation as with particle confinement, where the median-based background estimate locally approaches the value of background plus particle, thus reducing apparent particle contrasts in the ratiometric movie. For this reason, we recommend to perform MSPT experiments at localized particle densities ideally below 0.7 μm^{-2}.

As we were interested in quantifying the oligomer distribution of MinD at protein densities beyond this limit, we could not assume a simple Gaussian mixture model underlying the mass distributions in Figs. 2e/g, considering the observed distribution shape changes in our simulation. Fortunately, we found in a simulation with mixtures of particles with different contrasts that their respective peaks kept a particle-density-specific shape that was similar to the shape for the individual species at that particle density (Supplementary Fig. 10). Accordingly, we could use the simulated numerical form of a species mass distribution at a given particle density to fit a linear combination of these distributions with varying amplitudes to describe the mass distribution of a species mixture and extract their relative abundances (see Supplementary Fig. 11 for a fit of simulated data and Fig. 2e-h for a fit of our experimental data).

While this approach enables the deconvolution of complex mass distributions such as those observed for MinD at higher particle densities, where the underlying populations cannot be resolved experimentally, the remaining caveat is that this approach does not improve the particle detection efficiency itself. Hence, the abundance of small protein complexes close to the detection limit such as MinD dimers tend to be underestimated at high particle densities (Supplementary Figs. 10 and 11).

Supplementary Tables

Supplementary Table 1 Particle detection and trajectory linking parameters for MSPT measurements.

**Particle detection parameters**		**Trajectory linking parameters (Trackpy link_df function)**	
median half-size n	60	search_range	4 pixels
Laplace filter threshold	0.0007	memory	0 frames
Laplace filter sigma	1.5 pixels		
Local maximum filter size	7 pixels		
Refeyn PSF parameters	A12: -5.9360386376<br>W = 2.1436230397<br>S = 12.8930119585		

Supplementary Table 2 List of the molecular mass and the determined iSCAT contrast of standard proteins measured using the conventional MP landing assay.

**standard protein**	**used abbreviation**	**MW [kDa]**	**iSCAT contrast**
alcohol dehydrogenase	ADH	147.4	0.0053 ± 0.0002
bovine serum albumin	BSA	66.4	0.0025 ± 0.0002
TEV protease	TEV	28.6	0.0015 ± 0.0002
β-amylase	bAm	224.3	0.0080 ± 0.0010
protein A	prA	42.0	0.0019 ± 0.0002

Supplementary Table 3 List of the molecular mass and the determined iSCAT contrast of standard proteins measured using MSPT.

**standard protein**	**used abbreviation**	**expected MW [kDa]**	**iSCAT mass [kDa]**	**iSCAT contrast**
divalent streptavidin	Strep	55.2	52.2 ± 8.8	0.0022 ± 0.0004
divalent streptavidin with biotinylated aldolase	Strep-ALD	133.9 (2x ALD)<br>212.5 (4x ALD)	131.7 ± 13.0<br>193.0 ± 26.4	0.0049 ± 0.0005<br>0.0069 ± 0.0009
divalent streptavidin with biotinylated bovine serum albumin	Strep-BSA	124.5 (1x BSA)<br>193.8 (2x BSA)	127.0 ± 15.3<br>204.5 ± 22.2	0.0047 ± 0.0006<br>0.0072 ± 0.0008
divalent streptavidin with biotinylated protein A	Strep-prA	101.9	111.9 ± 15.9	0.0042 ± 0.0006
tetravalent streptavidin	-	55.2	55.1 ± 1.2	0.0023 ± 0.0001

Supplementary Table 4 List of all plasmids that were used for the characterization of the MinDE mechanism through MSPT.

**vector**	**protein construct**	**source**
pET28a-MinE-His	MinE-His	[4]
pET28a-His-MinD-MinE	MinD	[5]
pET28a-MinD(D40A)-MinE	MinD(D40A)	[6]
pET21a-Streptavidin-Alive	Streptavidin-Alive	[7]; Addgene plasmid #20860
pET21a-Streptavidin-Dead	Streptavidin-Dead	[7]; Addgene plasmid #20859

Supplementary Table 5 Number of detected trajectories of simulated particles with varying diffusion coefficients. Videos of simulated particles were either analyzed with a median window of 121 or 1001 frames.

	Median window of 121 frames		Median window of 1001 frames	
D_t [µm^2/s]	N(trajectories)		N(trajectories)	
0.01	4,143	2,210	5,415	3,891
0.032	14,525	10,401	3,692	3,004
0.1	10,072	8,492	4,470	3,781
0.32	10,498	9,273	6,739	5,910
1.0	17,090	15,324	10,637	9,558
3.2	20,881	19,071	13,554	12,368
10.0	28,880	25,613	19,030	16,816

Supplementary Table 6 Number of detected trajectories of simulated MinD and MinD D40A particles with respect to the particle density.

	monomer	dimer	trimer	tetramer	pentamer	hexamer
particle density [µm^{-2}]	N(trajectories)					
**MinD**						
0.3	11,524	17,892	19,604	18,958	19,402	19,654
0.5	26,531	52,722	55,829	49,248	57,528	57,868
0.6	15,895	59,883	65,019	52,919	66,897	66,872
0.7	4,752	73,411	63,690	60,632	62,524	65,148
0.8	-	103,375	66,002	73,141	63,726	64,981
0.9	-	144,803	84,756	82,659	83,495	81,390
1.0	-	159,423	101,707	74,458	101,969	102,317
1.1	-	123,062	99,319	49,996	99,241	102,678
1.2	-	59,992	72,792	22,537	71,813	75,321
1.3	-	18,013	37,098	6,119	34,951	36,882
**MinD D40A**						
0.3	11,524	18,938	5,905	17,100	5,585	6,014
0.5	26,531	59,486	16,059	49,522	14,765	15,505
0.6	15,895	74,810	15,600	52,183	14,632	15,552
0.7	4,752	75,636	14,442	50,917	15,464	16,591
0.8	-	79,327	19,142	62,124	19,170	18,976
0.9	-	95,539	24,806	76,452	22,748	21,562
1.0	-	101,936	27,228	85,122	22,984	22,374
1.1	-	79,968	25,654	78,347	20,450	21,985
1.2	-	41,960	18,553	52,117	14,528	16,721
1.3	-	14,680	9,745	26,031	6,868	8,480

Supplementary Table 7 Number of detected trajectories of simulated particles with respect to the particle density. The simulation was set up with particles that had theoretical masses corresponding to a 1:1 mixture of MinD monomers and dimers, a 1:1 mixture of MinD dimer and tetramer, and a 1:1:1 composition of particles with the MW of a MinD monomer, dimer and tetramer.

	**monomer/dimer**	**dimer/tetramer**	**monomer/dimer/tetramer**
**Particle density [µm^{-2}]**	**N(trajectories)**		
0.1	81,389	83,453	71,449
0.2	84,518	87,634	68,765
0.3	92,679	89,402	81,044
0.4	94,099	105,408	92,079
0.5	71,475	112,960	77,079
0.6	34,918	100,613	44,770
0.7	9,714	62,044	16,675
0.8	1,738	24,813	3,722

Supplementary Movies

Supplementary Movie 1 - Comparison of processed movies showing a single diffusing particle of biotin-aldolase bound to a biotinylated bilayer via divalent streptavidin. Left: processing strategy typically used for mass photometry (sliding mean, navg = 5, Supplementary Fig. 1a and b). Right: new processing strategy using a sliding median as background estimate (median half-size = 60 frames, Supplementary Fig. 1c). Scale bar: 1 μm. Interferometric scattering contrast range: black = 0.01; white = −0.008.

Supplementary Movie 2 - Exemplary movies showing standard proteins diffusing on a biotinylated bilayer attached via divalent streptavidin. Upper left: Divalent streptavidin alone. Upper right: biotin-BSA-streptavidin. Lower left: biotin-protein A-streptavidin. Lower right: biotin-aldolase-streptavidin. Image processing median half-size = 60 frames. Scale bar: 1 μm. Interferometric scattering contrast range: black = 0.004; white = −0.003.

Supplementary Movie 3 - Exemplary movies showing MinD (top) and MinDE (bottom) complexes diffusing on a bilayer. Solution concentrations: top - 100 nM MinD; bottom – 100 nM MinD, 100 nM MinE. Image processing median half-size = 60 frames. Scale bar: 1 μm. Interferometric scattering contrast range: black = 0.004; white = −0.003.

References

1. Evans, E. & Sackmann, E. Translational and rotational drag coefficients for a disk moving in a liquid membrane associated with a rigid substrate. *J. Fluid Mech.* **194**, 553–561 (1988).
2. Khmelinskaia, A., Mücksch, J., Petrov, E. P., Franquelim, H. G. & Schwille, P. Control of Membrane Binding and Diffusion of Cholesteryl-Modified DNA Origami Nanostructures by DNA Spacers. *Langmuir* **34**, 14921–14931 (2018).
3. Little, M. A. *et al.* Steps and bumps: Precision extraction of discrete states of molecular machines. *Biophys. J.* **101**, 477–485 (2011).
4. Glock, P. *et al.* Stationary patterns in a two-protein reaction-diffusion system. *ACS Synth. Biol.* **8**, 148–157 (2019).
5. Loose, M., Fischer-Friedrich, E., Ries, J., Kruse, K. & Schwille, P. Spatial regulators for bacterial cell division self-organize into surface waves in vitro. *Science* **320**, 789–792 (2008).
6. Heermann, T., Ramm, B., Glaser, S. & Schwille, P. Local Self-Enhancement of MinD Membrane Binding in Min Protein Pattern Formation. *J. Mol. Biol.* **432**, 3191–3204 (2020).
7. Howarth, M. *et al.* A monovalent streptavidin with a single femtomolar biotin binding site. *Nat. Methods* **3**, 267–273 (2006).
