# Supplementary material for: Mass-sensitive particle tracking to elucidate the membrane-associated MinDE reaction cycle
Source: Nat Methods. 2021 Oct 4;18(10):1239–46. doi: 10.1038/s41592-021-01260-x (PMC8490154; doi:10.1038/s41592-021-01260-x)
Supplement: Supplementary file 2 — Reporting Summary [file 41592_2021_1260_MOESM2_ESM.pdf]

nature research

Corresponding author(s): Prof. Petra Schwille
Dr. Nikolas Hundt

Last updated by author(s): Jul 21, 2021

Reporting Summary

Nature Research wishes to improve the reproducibility of the work that we publish. This form provides structure for consistency and transparency in reporting. For further information on Nature Research policies, see our Editorial Policies and the Editorial Policy Checklist.

Statistics

For all statistical analyses, confirm that the following items are present in the figure legend, table legend, main text, or Methods section.

n/a	Confirmed	
☐	☒	The exact sample size (*n*) for each experimental group/condition, given as a discrete number and unit of measurement
☐	☒	A statement on whether measurements were taken from distinct samples or whether the same sample was measured repeatedly
☒	☐	The statistical test(s) used AND whether they are one- or two-sided<br>*Only common tests should be described solely by name; describe more complex techniques in the Methods section.*
☒	☐	A description of all covariates tested
☐	☒	A description of any assumptions or corrections, such as tests of normality and adjustment for multiple comparisons
☐	☒	A full description of the statistical parameters including central tendency (e.g. means) or other basic estimates (e.g. regression coefficient) AND variation (e.g. standard deviation) or associated estimates of uncertainty (e.g. confidence intervals)
☒	☐	For null hypothesis testing, the test statistic (e.g. F, t, r) with confidence intervals, effect sizes, degrees of freedom and P value noted<br>*Give P values as exact values whenever suitable.*
☒	☐	For Bayesian analysis, information on the choice of priors and Markov chain Monte Carlo settings
☒	☐	For hierarchical and complex designs, identification of the appropriate level for tests and full reporting of outcomes
☒	☐	Estimates of effect sizes (e.g. Cohen's *d*, Pearson's *r*), indicating how they were calculated

Our web collection on statistics for biologists contains articles on many of the points above.

Software and code

Policy information about availability of computer code

Data collection	AquireMP (Refeyn Ltd., v2.3)
Data analysis	DiscoverMP (version 2.1.0, Refeyn Ltd.); Python version 3.8.5 - 1D kernel-density estimation with KDEpy (version 1.0.10) and 2D with fastkde (version 1.0.14); MATLAB R2020a Update 6; Kalafut-Visscher algorithm kvsteps function implemented by Max A. Little (http://www.maxlittle.net/software/steps_bumps_toolkit.zip)

For manuscripts utilizing custom algorithms or software that are central to the research but not yet described in published literature, software must be made available to editors and reviewers. We strongly encourage code deposition in a community repository (e.g. GitHub). See the Nature Research guidelines for submitting code & software for further information.

Data

Policy information about availability of data

All manuscripts must include a data availability statement. This statement should provide the following information, where applicable:

- Accession codes, unique identifiers, or web links for publicly available datasets
- A list of figures that have associated raw data
- A description of any restrictions on data availability

Microscopy raw data and tables of detected and linked particles have been deposited in the Max Planck data service Edmond under the following DOI: https://dx.doi.org/10.17617/3.74.

Field-specific reporting

Please select the one below that is the best fit for your research. If you are not sure, read the appropriate sections before making your selection.

☒ Life sciences ☐ Behavioural & social sciences ☐ Ecological, evolutionary & environmental sciences

For a reference copy of the document with all sections, see nature.com/documents/nr-reporting-summary-flat.pdf

Life sciences study design

All studies must disclose on these points even when the disclosure is negative.

Sample size	Sample size can be either defined as single particle/trajectory numbers or number of independent repeats of the same experiment. Particle numbers were very high for individual experiments and provided statistics to clearly determine molecule population behaviour. To show that this population behaviour was reproducible, 3 independent repeats of each experiment were considered sufficient, since deviations between repeats were low.
Data exclusions	No data was excluded.
Replication	Three independent experiments for all measurements. All replicates were used since none deviated significantly.
Randomization	Not applicable. No clinical trials involved. Single molecules provide intrinsically randomised data because their properties are unknown prior to their analysis and no data was excluded.
Blinding	Blinding was not required, since no data was excluded and the analysis routines were completely automated. Hence, analysis was intrinsically without user-bias.

Reporting for specific materials, systems and methods

We require information from authors about some types of materials, experimental systems and methods used in many studies. Here, indicate whether each material, system or method listed is relevant to your study. If you are not sure if a list item applies to your research, read the appropriate section before selecting a response.

Materials & experimental systems

n/a	Involved in the study	
☒	☐	Antibodies
☒	☐	Eukaryotic cell lines
☒	☐	Palaeontology and archaeology
☒	☐	Animals and other organisms
☒	☐	Human research participants
☒	☐	Clinical data
☒	☐	Dual use research of concern

Methods

n/a	Involved in the study	
☒	☐	ChIP-seq
☒	☐	Flow cytometry
☒	☐	MRI-based neuroimaging
